# Cdc42 regulates Cdc42EP3 function in cancer-associated fibroblasts

**DOI:** 10.1080/21541248.2016.1194952

**Published:** 2016-06-01

**Authors:** Aaron J. Farrugia, Fernando Calvo

**Affiliations:** Tumour Microenvironment Team, Division of Cancer Biology, Institute of Cancer Research, London, UK

**Keywords:** BORG, cancer-associated fibroblasts, Cdc42EP3, cytoskeleton, Rho GTPases, septin

## Abstract

Rho family GTPases such as Cdc42 are key regulators of essential cellular processes through their effects on cytoskeletal dynamics, signaling and gene expression. Rho GTPases modulate these functions by engaging a wide variety of downstream effectors. Among these effectors is the largely understudied Cdc42EP/BORG family of Cdc42 effectors. BORG proteins have been linked to actin and septin regulation, but their role in development and disease is only starting to emerge. Recently, Cdc42EP3/BORG2 was shown to coordinate actin and septin cytoskeleton rearrangements in cancer-associated fibroblasts (CAFs). Interestingly, Cdc42EP3 expression potentiated cellular responses to mechanical stimulation leading to signaling and transcriptional adaptations required for the emergence of a fully activated CAF phenotype. These findings uncover a novel role for the BORG/septin network in cancer. Here, we demonstrate that Cdc42EP3 function in CAFs relies on tight regulation by Cdc42.

Cancer-associated fibroblasts (CAFs) are stromal cells that can influence tumor progression, dissemination and response to therapy through remodeling of the extracellular matrix (ECM) and signaling to cancer, endothelial and immune cells.^1^ One major feature of CAFs is their dramatic rearrangement of cytoskeletal networks. CAFs are characterized by the presence of enhanced contractile actin stress fibers, upregulation of cytoskeletal components such as smooth muscle actin (αSMA) and palladin, and stronger focal adhesions.^2-4^ These changes impinge on the capacity of CAFs to exert force and generate invading structures, and directly affect their ability to remodel the ECM and enable subsequent cancer cell invasion.^3-6^ Force-mediated ECM remodeling by CAFs also enhances tissue stiffness that can promote malignancy by activating mechano-transduction signaling pathways that modulate cancer cell growth, survival and migration.^7^ In addition, increased actomyosin contractility in CAFs enables fibroblasts to respond to changes in matrix stiffness and leads to activation of the transcriptional regulator YAP, which is critical for maintaining a CAF phenotype.^3^

Rho family GTPases are key regulators of cytoskeletal organization through their effects on actin assembly, actomyosin contractility and microtubules, among others.^8^ Rho GTPases are particularly relevant in CAFs. RhoA activity is generally higher in CAFs compared to normal fibroblasts (NFs), and mediates the phosphorylation of myosin light chain 2 (MLC2) at S19 and/or T18 via Rho-kinases ROCK1 and ROCK2. This leads to MLC2 activation, a highly contractile cytoskeleton and matrix remodeling.^5,6^ In contrast, Rac1 is mainly inactivated in CAFs.^6^ Importantly, modulating the balance of RhoA/Rac1 signaling has been shown to significantly affect CAF functions.^3,5,6^ In addition, Cdc42 signaling through its effectors MRCKα and β can also generate actomyosin contractility, through direct phosphorylation of MLC2^9-11^ and activation of Cdc42 can also promote the formation of matrix-degrading invadopodia in CAFs leading to enhanced tumor cell invasion.^4^

Based on the well-established links between cytoskeletal organization and CAF functions, we recently conducted a study to identify novel regulators of CAFs by performing an RNAi loss-of-function screen of cytoskeleton-related factors.^12^ Using NFs and CAFs isolated from a murine model of breast cancer, the transgenic FVB/n strain expressing the polyoma middle T antigen oncogene under the mouse mammary tumor virus promoter (MMTV-PyMT),^3^ we compared their cytoskeletal organization and observed that this model recapitulates most of the characteristics of CAFs. Compared to NFs, CAFs had enhanced F-actin stress fibers containing active MLC2, abundant paxillin-positive focal adhesions and up-regulation of αSMA (Fig. 1A). We also explored other cytoskeletal networks and observed no obvious differences in the organization of intermediate filaments and microtubules between NF and CAFs (Fig. 1B). Septins are increasingly recognized as cytoskeletal components, due to their ability to (i) assemble into oligomeric complexes (such as the well characterized SEPT2/6/7 heterotrimer); (ii) form higher-order structures such as filaments and rings; and (iii) associate with cellular membranes, actin filaments and microtubules.^13,14^ Interestingly, our analyses showed that CAFs presented more extensive septin networks (Fig. 1C) that generally co-aligned with actin stress fibers (Fig. 1D), describing a novel cytoskeletal characteristic of CAFs.

**Figure 1.**
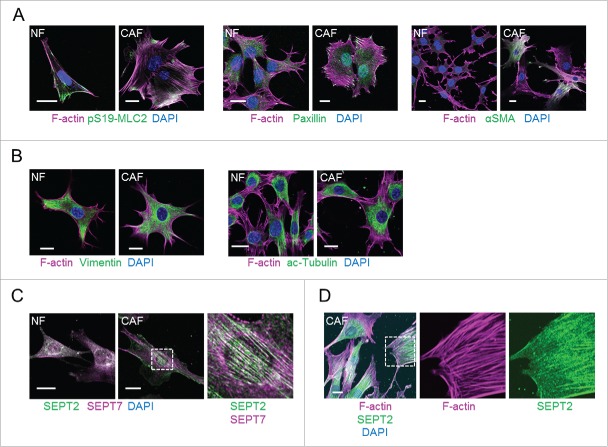
Cytoskeletal networks in normal mammary fibroblasts (NFs) and MMTV-PyMT derived breast CAFs (CAFs). (A) Left panels show images of F-actin (magenta), pS19-MLC2 (green) and DAPI (blue). Middle panels show F-actin (magenta), paxillin (green) and DAPI (blue) staining. Right panels show F-actin (magenta), αSMA (green) and DAPI (blue) staining. Scale bars represent 20 μm. (B) Left panels show images of F-actin (magenta), vimentin (green) and DAPI (blue). Right panels show F-actin (magenta), acetylated tubulin (green) and DAPI (blue) staining. Scale bars represent 20 μm. (C) Images show SEPT2 (green), SEPT7 (magenta) and DAPI (blue) staining. A zoom up area is shown for CAFs. Scale bars represent 20 μm. (D) Images show staining of F-actin (magenta), SEPT2 (green) and DAPI (blue) in CAFs. A zoom up area for F-actin and SEPT2 is shown. Scale bar represents 20 μm.

Our screen identified Cdc42 effector protein 3 (Cdc42EP3)/BORG2 as a key regulator of CAFs.^12^ Using biochemical approaches and super-resolution microscopy we demonstrated that Cdc42EP3 can independently bind F-actin and septins, localizes at the interface between F-actin fibers and SEPT2 filaments and stabilizes both networks in CAFs. In addition, we observed a particular pattern of SEPT2 and Cdc42EP3 localization between stress fibers suggesting that they could provide mechanical support for the contractile actin cytoskeleton. In agreement, depletion of Cdc42EP3 or SEPT2 led to the full disruption of actomyosin fibers and loss of focal adhesions. The main consequence was a drastic functional inactivation of CAFs, as Cdc42EP3-depleted CAFs presented reduced matrix remodeling, cancer cell invasion, angiogenesis and tumor growth promoting abilities, a phenotype that was mimicked by SEPT2-depletion.

Gain-of-function analyses demonstrated that ectopic expression of Cdc42EP3 in NFs could induce the formation of F-actin fibers and septin filaments, reminiscent of CAF-like cytoskeletal rearrangements. Interestingly, direct binding of Cdc42EP3 to both F-actin and septins was required to drive both networks into a filamentous state. We observed that a Cdc42-binding defective mutant of Cdc42EP3 (Cdc42EP3-IS) could not interact with actin in co-immunoprecipitation assays.^12^ Here, we further examined the localization and activity of this mutant and the role of Cdc42 in modulating Cdc42EP3 functions in NFs and CAFs.

When expressed in NFs, Cdc42EP3-IS presented a diffuse cytosolic localization, in striking contrast to the filamentous appearance of wild-type Cdc42EP3 (Fig. 2A and B). In addition, this Cdc42-binding defective mutant was no longer able to induce F-actin and septin reorganization. Cdc42EP3-IS mislocalization was also evident in CAFs, which present increased basal levels of F-actin and filamentous septin, thus ruling out any requirement of F-actin/filamentous septin structures for the correct positioning of this mutant (Fig. 3A and B). On the contrary, Cdc42EP3-IS appeared to act as a dominant-negative mutant as both actin stress fibers and particularly SEPT2 filaments were amply diminished in CAFs following Cdc42EP3-IS transfection (Fig. 3A and B). In agreement, expression of Cdc42EP3-IS in CAFs resulted in reduced pS19-MLC2 staining and fewer paxillin-positive focal adhesions (Fig. 3C). This phenotype was very similar to that observed following Cdc42EP3 depletion.^12^

**Figure 2.**
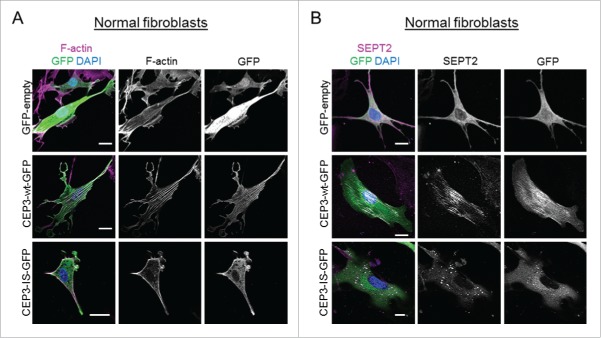
A Cdc42-defective binding mutant of Cdc42EP3 presents a diffuse pattern of cytosolic localization and does not induce actin and septin rearrangements in normal fibroblasts. (A) Panels show GFP (green), F-actin (magenta) and DAPI (blue) staining of normal fibroblasts following transfection with GFP or GFP-tagged wild-type (wt) or IS mutant (IS) Cdc42EP3 (CEP3) proteins. The grayscale panels show individual channels for F-actin and GFP. Scale bars, 25 μm. (B) Panels show GFP (green), SEPT2 (magenta) and DAPI (blue) staining of normal fibroblasts following transfection with GFP or GFP-tagged wild-type (wt) or IS mutant (IS) Cdc42EP3 (CEP3) proteins. The grayscale panels show individual channels for F-actin and GFP. Scale bars, 25 μm.

**Figure 3.**
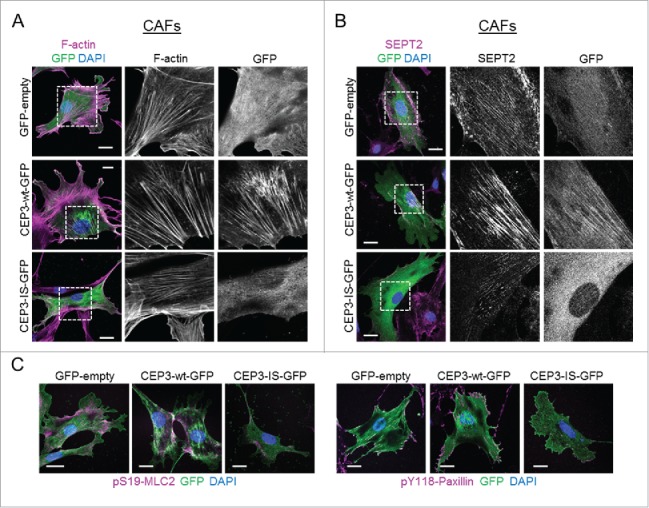
A Cdc42-defective binding mutant of Cdc42EP3 acts as a dominant-negative when expressed in cancer-associated fibroblasts (CAFs). (A) Panels show GFP (green), F-actin (magenta) and DAPI (blue) staining of cancer-associated fibroblasts (CAFs) following transfection with GFP or GFP-tagged wild-type (wt) or IS mutant (IS) Cdc42EP3 (CEP3) proteins. The grayscale panels show individual channel magnifications of perinuclear areas. Scale bars, 25 μm. (B) Panels show GFP (green), SEPT2 (magenta) and DAPI (blue) staining of CAFs following transfection with GFP or GFP-tagged wild-type (wt) or IS mutant (IS) Cdc42EP3 (CEP3) proteins. The grayscale panels show individual channel magnifications of perinuclear areas. Scale bars, 25 μm. (C) Left panels show images of GFP (green), pS19-MLC2 (magenta) and DAPI (blue) staining of CAFs following transfection with GFP or GFP-tagged wild-type (wt) or IS mutant (IS) Cdc42EP3 (CEP3) proteins. Right panels show GFP (green), pY118-Paxillin (magenta) and DAPI (blue) staining of CAFs following transfection with GFP or GFP-tagged wild-type (wt) or IS mutant (IS) Cdc42EP3 (CEP3) proteins. Scale bars represent 25 μm.

To further investigate the role of Cdc42 in regulating Cdc42EP3 functions, we monitored Cdc42EP3 localization and actin/septin rearrangements in CAFs after expression of Cdc42-N17 and Cdc42-V12, dominant negative and constitutively active mutant versions of Cdc42, respectively. Inhibiting Cdc42 function by transient expression of the dominant negative Cdc42-N17 protein resulted in a loss of filamentous Cdc42EP3 structures and reduced F-actin stress fibers and filamentous SEPT2 (Fig. 4A and B). However, boosting Cdc42 activity by transient expression of the constitutively active mutant Cdc42-V12 did not potentiate Cdc42EP3 activity. On the contrary, expression of Cdc42-V12 sequestered Cdc42EP3 to Cdc42-V12-rich vesicles and resulted in a complete loss of filamentous Cdc42EP3 (Fig. 4A and B). Cdc42-V12 expression also led to reduced levels of perinuclear F-actin fibers and SEPT2 filaments. These findings are in agreement with previous studies showing that the function of the closely related protein Cdc42EP5 over septins is negatively regulated by constitutively active Cdc42.^15^ In general, these data suggest that the coordinating function of Cdc42EP3 over F-actin and septins depends on Cdc42, which regulates the correct positioning of Cdc42EP3 and its ability to form filaments. However, this process relies on the ability of Cdc42 to cycle between active and inactive states. If Cdc42 is locked in an active state (like in the case of the constitutively active mutant Cdc42-V12), Cdc42EP3 is also severely affected. Whether Cdc42-V12 localization is not ideal for the correct positioning of Cdc42EP3 or whether the transition between active/inactive Cdc42 is required for Cdc42EP3 functions (i.e. for releasing it and allowing other essential molecular interactions) are details still to be determined.

**Figure 4.**
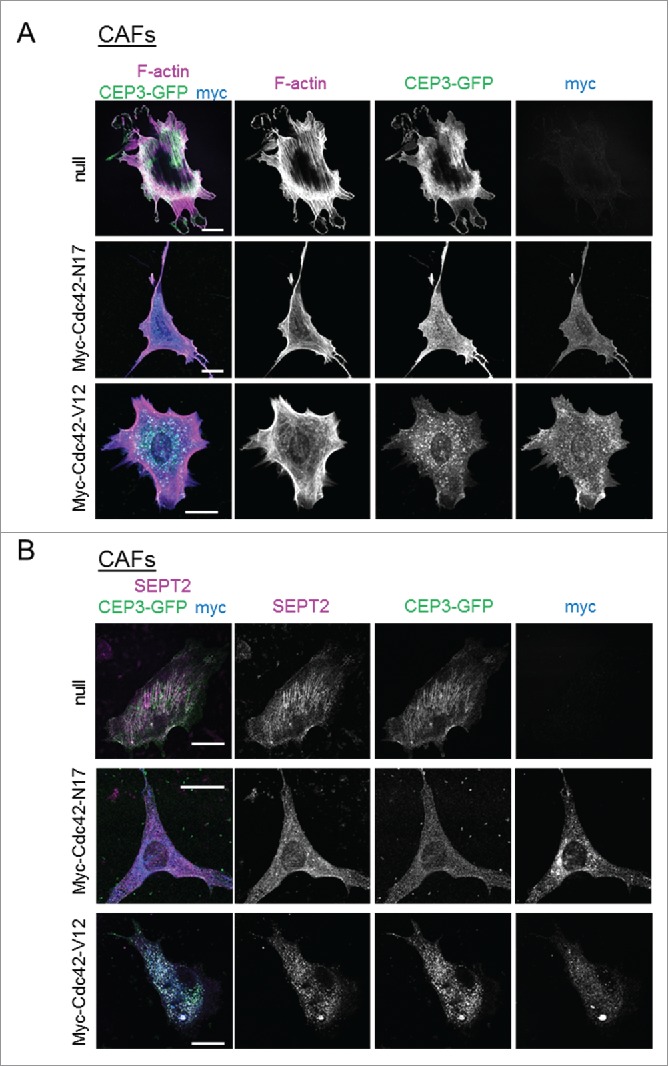
Modulating the activity of Cdc42 in CAFs affects Cdc42EP3 localization and septin and F-actin organization. (A) Panels show GFP (green), F-actin (magenta) and myc (blue) staining of cancer-associated fibroblasts (CAFs) stably expressing Cdc42EP3-GFP following transfection with empty vector, myc-Cdc42-N17 or myc-Cdc42-V12. The grayscale panels show individual channels as indicated. Scale bars, 25 μm. (B) Panels show GFP (green), SEPT2 (magenta) and myc (blue) staining of cancer-associated fibroblasts (CAFs) stably expressing Cdc42EP3-GFP following transfection with empty vector, myc-Cdc42-N17 or myc-Cdc42-V12. The grayscale panels show individual channels as indicated. Scale bars, 25 μm.

Septin filament assembly can follow an actin template by means of adaptor proteins.^16^ Our study demonstrated that Cdc42EP3 depletion reduced the filamentous septin network in CAFs,^12^ an effect that was also detected after blocking stress fiber formation by depleting the well-known actin remodeling proteins DIAPH1 and DIAPH3.^17^ Recently, it has been shown that SEPT9 can bind F-actin and promote the cross-linking of preassembled actin filaments,^18,19^ underlying the notion of a functional inter-relationship between both networks. Our data suggested that Cdc42EP3 may act as a ‘molecular bridge’ between septin filaments and F-actin fibers that reinforces both networks.^12^ In CAFs, this interaction may allow the “template function” of actin needed for septin polymerization, as well as potentiating the F-actin cross-linking activity of septins. Still to be determined is whether Cdc42EP3 presents intrinsic septin and F-actin polymerization/cross-linking activities that could also explain these observations.

We also observed that Cdc42EP3 and SEPT2 organization changes in response to substrate stiffness in CAFs.^12^ Moreover, Cdc42EP3 and septin filaments were required and in part sufficient for CAFs to respond to mechanical stimulation and activate the key regulator YAP, indicative of a novel molecular role for these proteins in mechano-transduction. Our data suggested that Cdc42EP3 and septins perform this function by reinforcing the contractile actin cytoskeleton, thus allowing for increased isometric tension in CAFs. Further studies are needed to determine whether Cdc42EP3 and septins can regulate mechano-transduction *per se* or via alternative actomyosin-independent mechanisms.

These properties and functions may not be exclusive of Cdc42EP3 in CAFs. Filamin A (FLNA) is a cytoskeletal protein that organizes filamentous actin in networks and stress fibers,^20^ binds to SEPT9^21^ and participates in mechano-sensing.^22^ Anillin (ANLN) is an adaptor protein that mediates septin assembly following an actin template during cytokinesis.^16^ Accordingly, perturbing ANLN function or septin organization results in attenuation of actin stress fibers in NIH3T3 fibroblasts.^16^ Both FLNA and ANLN were found to be upregulated in CAFs and were evaluated in our RNAi screen.^12^ Only FLNA depletion seemed to affect CAF functionality, albeit to a lesser extent than Cdc42EP3. It will be interesting to further investigate whether FLNA plays similar roles to Cdc42EP3 in CAFs or whether it coordinates alternative mechanisms.

Other related proteins may operate in a similar fashion to Cdc42EP3. Cdc42EP3 is a member of the BORG/Cdc42EP family of Cdc42 effectors, which comprises of 5 members (Fig. 5A).^23,24^ Noteworthy, Cdc42EP3 was the only BORG protein that presented differential expression between NFs and CAFs;^12^ this may be different in other systems. BORG proteins share a Cdc42/Rac interactive binding (CRIB) motif that allows the interaction with active Cdc42 and RhoQ/TC10 but not RhoA or Rac1.^23-25^ The defining characteristic of BORG proteins is the presence of 3 BORG specific domains (BD1-3). The BD3 domain is present in all members and mediates the interaction between BORG proteins and septin complexes (Fig. 5B),^15,26^ allowing BORGs to regulate septin organization in mammalian cells.^15,23^ In addition, ectopic expression of BORGs leads to changes in cell shape and the formation of F-actin containing structures and pseudopodia,^23-25^ suggesting that other BORG proteins may also bind and modulate F-actin networks. In endothelial cells, Cdc42EP1 in conjunction with septins, promotes angiogenesis by regulating persistent directional migration through spatial control of actomyosin contractility,^27^ a function reminiscent of the one performed by Cdc42EP3 in CAFs. We observed that the binding of Cdc42EP3 to F-actin was dependent on a specific motif that is not widely conserved across the family (Fig. 5C). This suggests that BORGs may modulate actin networks by alternative mechanisms. One possibility is that the activity of BORGs over actin may also depend on their modulatory role over septins, that may affect F-actin polymerization directly^18^ or via adaptor proteins.^16^ Alternatively, this activity may be mediated in other BORGs by different domains or via alternative modes of action that still need to be fully characterized. For example, PKC-mediated phosphorylation reduces the affinity of Cdc42EP4 to Cdc42 in MCF-10A cells. This allows binding to the Rac-GEF ARHGEF17, resulting in Rac1 activation, altered actin containing structures and cell migration.^28^ In addition, ectopic expression of Cdc42EP5 in NIH3T3 fibroblasts leads to RhoA inhibition and loss of stress fibers in a mechanism that is independent of Cdc42 binding.^23^ We find these functional interactions particularly intriguing as they suggest that, despite not having any direct link with other Rho GTPases,^23^ BORG proteins can still modulate their activity and functions.

**Figure 5.**
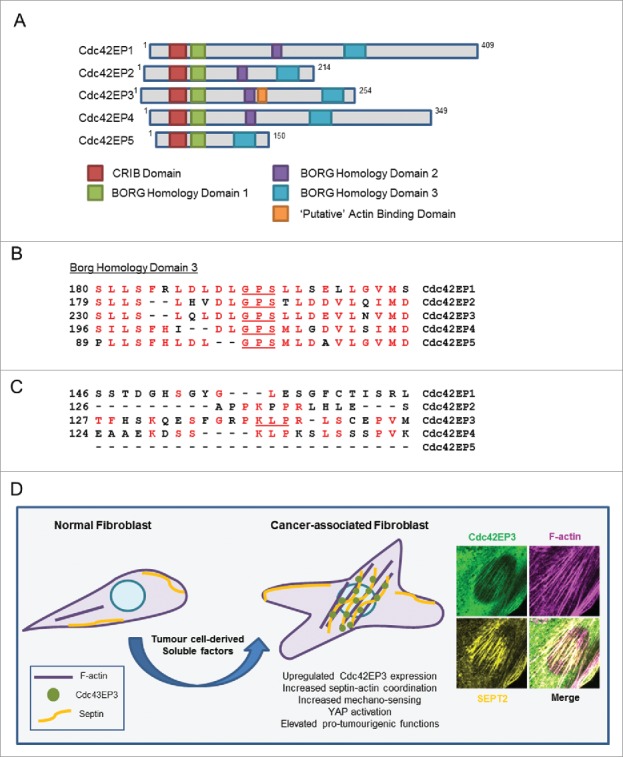
The Cdc42EP/BORG family of Cdc42 effectors. (A) Schematic showing the different BORG protein domains. (B) The BORG homology 3 (BD3) domains of Cdc42EP1-5, with conserved residues in red. Key residues within that region are underlined. (C) Direct alignments of the putative actin binding domain of Cdc42EP3, in comparison with other BORGs. This domain is absent in Cdc42EP5. Conserved residues are in red, key residues are underlined. (D) Model outlining the role of Cdc42EP3 and septins in the emergence of CAFs. Tumor cell-derived soluble factors promote the upregulation of Cdc42EP3 expression in NFs. This results in an altered cytoskeletal network with increased septin-actin cohesion and isomeric tension. As a result, cellular responses to mechanical stimulation are potentiated, leading to signaling and transcriptional adaptations (e.g. YAP activation) required for the emergence of a fully activated CAF phenotype. (Right) Immunofluorescence of the perinuclear region in CAFs, showing co-localization of Cdc42EP3 (green), F-actin (magenta) and SEPT2 (yellow).

Interestingly, Cdc42EP3 was required for the transition from a NF to a highly contractile CAF. We also observed that Cdc42EP3 upregulation occurred early during fibroblast activation in cancer in response to multiple factors, including HGF, TGFβ, and SDF-1α. Both chemical and mechanical stimulation are suggested to be responsible for the pathological activation of fibroblasts.^29^ Our model (Fig. 5D) suggests that Cdc42EP3 is located at a key node linking both activating processes. We propose that Cdc42EP3 expression induced by cancer cells or inflammatory signals in the stroma of pre-malignant lesions would sensitize fibroblasts to respond to changes in the physical environment. These changes would enable the subsequent activation of mechano-transduction signaling pathways needed for the emergence of a fully activated CAF.

The linking and reinforcement of actin and septin networks may be also critical in other contexts. In cancer, actin polymerization and actomyosin contractility are key events in migratory tumor cells and metastasis.^30^ The activation of mechano-responsive signaling pathways can also promote malignancy by enhancing cancer cell growth, survival and migration.^7^ In addition, SEPT9 has been shown to be essential for pseudopod protrusion and tumor cell migration and invasion by promoting the cross-linking of lamellar stress fibers and the stability of nascent focal adhesions.^18,31^ In a physiological context, septins have been shown to regulate lymphocyte trafficking in confined tissues by tuning actomyosin forces during motility.^32^ Whether BORGs also play a role in these settings is an interesting possibility worth exploring.

To conclude, our study demonstrated that CAFs represent a powerful tool to investigate cytoskeletal regulation in a biologically relevant setting. We uncovered a critical role for the coordination of actin and septin networks in CAFs. Disrupting this coordinated interaction prevented the activation of mechano-sensing signaling pathways, including paxillin, Src and YAP. CAFs have been shown to participate in many of the hallmarks of cancer and are being suggested as potential therapeutic targets.^1^ Preventing F-actin and septin coordination largely impacts the pro-tumorigenic properties of CAFs, diminishing their force-mediated matrix remodeling, cancer cell invasion, angiogenesis, and tumor growth promoting abilities. We identified Cdc42EP3 as a critical regulator of these activities, through tight regulation by Cdc42. Whether targeted disruption of Cdc42EP3 function represents a therapeutic opportunity to deactivate CAFs, is an enticing possibility that merits further investigation.

## Materials and methods

### 

#### cDNAs

Murine Cdc42EP3 cDNAs have been previously described. Briefly, Cdc42EP3 cDNA from pCMV-Sport-6-Cdc42EP3 was subcloned into pEGFP.C1 backbone. pEGFP-Cdc42EP3 was used as a template to generate the mutant Cdc42EP3(IS-AA) using the GeneArt Site-Directed Mutagenesis PLUS kit (Life Technologies). Sequences of the degenerated oligonucleotides can be provided on request. GFP-Cdc42EP3 was also subcloned in the pCSII-IRES lentiviral system that was used to generate stable cells lines. Plasmids encoding myc-tagged Cdc42 N17 and V12 mutants were a gift from Erik Sahai (Crick Institute, London, UK).

#### Cell lines

Fibroblasts from normal mammary glands (NFs) and mammary carcinoma (CAFs) have been previously described.^3^ Briefly, normal mammary glands and mammary carcinoma tissue (12 week-old females) from MMTV-PyMT mice were dissected. Fibroblasts were isolated and immortalized with the HPV-E6 virus followed by selection on 2.5 μg/mL puromycin.^33^ Resulting populations were assessed for fibroblast and CAF marker expression and thoroughly characterized.^3^ All fibroblasts were cultured in DMEM (Invitrogen) 10% FBS, 1% insulin-selenium-transferrin (ITS). Cell lines stably expressing Cdc42EP3-GFP were generated by lentiviral infection.

#### Transfections

Fibroblasts were cultured in standard conditions and seeded at a density of 1.5 × 10^5^ cells/ml in a 6-well plate the day before transfection. cDNA transfection was performed using Lipofectamine 3000 reagent (Life Technologies) for all plasmids as per supplier's instructions.

### Immunofluorescence

Cells were usually fixed in 4% paraformaldehyde for 1 h. For the analysis of endogenous septins, cells were fixed in ice-cold methanol for 10 min. Cells were permeabilized by incubation in PBS 0.5% NP-40 (Sigma) at 4°C for 20 min (twice), in PBS 0.3% Triton 100 (Sigma) at room temperature (RT) for 20 min and in PBS 0.1% Triton 100 at RT for 15 min (twice). Samples were blocked for 60 min at RT (twice) in blocking solution: 4% BSA PBS 0.05% Tween20 (Sigma). Then, cells were incubated with primary antibody in blocking solution in a wet chamber overnight at 4°C. After 3 washes of 15 min in PBS, secondary antibody in blocking solution was added for 3 h. After 3 washes of 15 min in PBS, samples were mounted and analyzed using an inverted Zeiss LSM780 confocal microscope. Antibody description and working dilutions are: Acetylated-Tubulin (Abcam ab24610, 1:500), αSMA (Sigma A2547, 1:500), c-Myc (Cell Signaling 5605, 1:100), Paxillin (Transduction labs 510051, 1:100), Phospho-Paxillin Y118 (Invitrogen 44-722G, 1:100), Phalloidin-TRITC (Sigma P1951, 1:500), Phospho-MLC2 S19 (Cell Signaling, 3672, 1:100), SEPT2 (Proteintech 60075, 1:200), SEPT7 (Proteintech 13818, 1:200), Vimentin (Sigma, V2258, 1:200).

## Abbreviations

αSMA: smooth muscle actin, α
BD: BORG specific domain
BORG: binder of Rho GTPases
CAF: cancer-associated fibroblast
CRIB: Cdc42/Rac interactive binding
ECM: extracellular matrix
GEF: guanine nucleotide exchange factor
HGF: hepatocyte growth factor
MMTV-PyMT: mouse mammary tumor virus – polyoma-middle T antigen
NF: normal fibroblast
SDF-1α: stromal cell-derived factor 1 α
TGFβ: transforming growth factor β
